# Prevalence and management of chronic breathlessness in COPD in a tertiary care center

**DOI:** 10.1186/s12890-019-0851-5

**Published:** 2019-05-16

**Authors:** H. Carette, M. Zysman, C. Morelot-Panzini, J. Perrin, E. Gomez, A. Guillaumot, P. R. Burgel, G. Deslee, P. Surpas, O. Le Rouzic, T. Perez, A. Chaouat, N. Roche, F. Chabot, on behalf of the Initiatives BPCO (bronchopneumopathie chronique obstructive) Scientific Committee and Investigators, M. Zysman, P. R. Burgel, G. Deslee, P. Surpas, O. Le Rouzic, T. Perez, N. Roche, G. Brinchault-Rabin, D. Caillaud, P. Chanez, I. Court-Fortune, R. Escamilla, G. Jebrak, P. Nesme-Meyer, C. Pinet, Brigitte Risse

**Affiliations:** 1 Pulmonary Department, Nancy, France; 2 InsermU955, team 04, 8 rue du general Sarrail, 94000 Créteil, France; 3 GH Pitié-Salpêtrière, Respiratory and Intensive Care Medicine Department, Paris, France; 4 Respiratory and Intensive Care Medicine Department, Cochin Hospital, AP-HP and Paris Descartes University (EA 2511), Sorbonne Paris Cité, Paris, France; 5 Pulmonary Department, Maison Blanche University Hospital, INSERM U01250, Reims, France; 6 Centre médical de Bayère, 30, route du Vieux-Château, 69380 Charnay, France; 7 University Lille, CNRS, Inserm, CHU Lille, Institut Pasteur de Lille, U1019 - UMR 8204 - CIIL - Center for Infection and Immunity of Lille, F-59000 Lille, France

**Keywords:** Chronic obstructive pulmonary disease, Breathlessness, Opioid, Doctors ‘attitude

## Abstract

**Background:**

Breathlessness is the prominent symptom of chronic obstructive pulmonary disease (COPD). Despite optimal therapeutic management including pharmacological and non-pharmacological interventions, many COPD patients exhibit significant breathlessness. Chronic breathlessness is defined as breathlessness that persists despite optimal treatment of the underlying disease. Because of the major disability related to chronic breathlessness, symptomatic treatments including opioids have been recommended by several authors. The prevalence of chronic breathlessness in COPD and its management in routine clinical practice have been poorly investigated. Our aim was to examine prevalence, associated characteristics and management of chronic breathlessness in patients with COPD recruited in a real-life tertiary hospital-based cohort.

**Methods:**

A prospective study was conducted among 120 consecutive COPD patients recruited, in stable condition, at Nancy University Hospital, France. In parallel, 88 pulmonologists of the same geographical region were asked to respond to an on-line questionnaire on breathlessness management.

**Results:**

Sixty four (53%) patients had severe breathlessness (modified Medical Research Council scale≥3), despite optimal inhaled medications for 94% of them; 40% had undergone pulmonary rehabilitation within the past 2 years. The severity of breathlessness increased with increasing airflow limitation. Breathlessness was associated with increased symptoms of anxiety, depression and with osteoporosis. No relation was found with other symptoms, exacerbation rate, or cardiovascular comorbidities. Among the patients with chronic breathlessness and Hospitalized Anxiety and/or Depression score > 10, only 25% were treated with antidepressant or anxiolytic. Among the pulmonologists 46 (52%) answered to the questionnaire and expressed a high willingness to prescribe opioids forchronic breathlessness, which contrasted with the finding that none of these patients received such treatments against breathlessness.

**Conclusion:**

Treatment approaches to breathlessness and associated psychological distress are insufficient in COPD. This study highlights underuse of pulmonary rehabilitation and symptomatic treatment for breathlessness.

**Electronic supplementary material:**

The online version of this article (10.1186/s12890-019-0851-5) contains supplementary material, which is available to authorized users.

## Background

Breathlessness is the first reported symptomin patients with chronic obstructive pulmonary disease (COPD) [1]. Chronic breathlessness is defined as severe breathlessness (modified Medical Research Council dyspnea (mMRC) scale ≥3) that persists despite optimal treatment of the underlying pathology, resulting in disability [2, 3], which is very similar to refractory breathlessness [4]. COPD medications (long acting beta 2 agonists, LABA, long acting anti muscarinic, LAMA, and inhaled corticosteroids, ICS) and non-pharmacologic interventions (especially increase in physical activity or pulmonary rehabilitation) may be insufficiently effective to relieve this symptom. Breathlessness-specific therapies that alleviate refractory breathlessness and improve exercise capacity are needed to enhance health outcomes in advanced COPD [5]. Previous studies have demonstrated the efficacy of low-dose systemic opioids for chronic breathlessness [6–10], even if there is conflicting data [11]. In addition, based on current scientific knowledge, there is still no other effective medication. However, there is an important reluctance to prescribe this treatment [11–13].

In the present study performed in a real life cohort of patients suffering from COPD (the Initiatives BPCO French cohort, iBPCO cohort), our aim was to examine the characteristics associated with and prevalence of chronic breathlessness in patients with COPD recruited in a tertiary hospital-based cohort. We also investigated the management of chronic breathlessness regarding the use of opioids among pulmonologists of the same region. This study further evaluated the knowledge of pulmonologists about treatments against breathlessness, and their willingness to prescribe morphine for the treatment of severe or chronic breathlessness.

## Methods

This was a prospective descriptive cohort study. INITIATIVES BPCO is a national cohort of patients with spirometry-confirmed COPD recruited by respiratory physicians from tertiary care university hospital centres, as previously described [14]. The present study population concerns 120 first consecutive stable COPD patients included in the cohort from June 2016 to February 2017 in the Department of Respiratory Medicine, University Hospital of Nancy (Lorraine region, France). This article focuses on breathlessness with an extended interest on drugs such as morphine or benzodiazepines to alleviate this symptom.

### Patients

The following data were collected at inclusion: medical and smoking history, occupational exposures, breathlessness, exacerbations in the previous year, comorbidities including osteoporosis, body mass index (BMI), St George Respiratory Questionnaire (SGRQ) total score, hospital anxiety and depression score to screen anxious and depressive symptoms (HAD score anxiety and/or depression > 10 corresponding to high probability of symptoms of anxiety and/or depression) [15], activity daily living (ADL) [16], post-bronchodilator spirometry, pulmonary rehabilitation in the last 2 years, and medications. Cardiovascular disease was defined by declarative way, specialized cardiovascular follow-up or specific cardiovascular medications (at least beta-blockers or angiotensin-converting enzyme inhibitors).

### Definition of severe and chronic breathlessness

Severe breathlessness was defined by mMRC scale ≥3. We defined chronic breathlessness as severe breathlessness that persists more than 3 months [3] despite optimal treatment of the underlying disease (considered as such when recommended medications according to GOLD recommendations were prescribed: LABA or LAMA or LABA and LAMA +/− ICS [2, 17]), and that results in disability [2]. We distinguished chronic breathlessness despite pulmonary rehabilitation within the past 2 years (supervised exercise training and therapeutic education) from chronic breathlessness without pulmonary rehabilitation.

### Pulmonologist questionnaire (Additional file 1: Table S1)

All pulmonologists from the Lorraine region were invited to complete an anonymous and confidential on-line questionnaire from 2017/02/27 to 2017/03/27, which was developed for this study specifically. This questionnaire explored the willingness and experience in prescribing pulmonary rehabilitation and medications including opioids, anxiolytic and antidepressant in patients with COPD and breathlessness treated as out-patients, or in hospital, or at the end of life.

### Statistical analysis

Qualitative variables were described in numbers and percentages. Quantitative variables were described by mean and standard deviation or median and inter-quartile range. Differences in clinical characteristics were assessed using chi-square tests or Fisher’s exact tests, as appropriate, for discrete variables, and Wilcoxon or Kruskal–Wallis tests for quantitative variables.Relationships between different variables were examined with the Pearson correlation test.A value of *p* ≤ 0.05 was considered statistically significant. The analysis of the results was carried out with SAS® 9.2 statistical software.

### Ethics approval

The study was approved by the Ethics Committee of Versailles, France (number: 04–479). All patients provided written consent.

## Results

### Patients

Main characteristics of patients (*n* = 120) are provided in Table 1. In the overall population, the median FEV1 was 47 (26–60) % of predicted values, the median BMI 29 (21–31) Kg.m^− 2^, and the median smoking 40 (28–51) pack/year (PA). Sixty four (53%) patients had severe breathlessness, including 39 (32%) patients with mMRC = 3 and 25 (21%) patients with mMRC = 4. Ninety four percent of patients with severe dyspnea were currently receiving inhaled medications considered as optimal (i.e., two bronchodilators or a combination of long acting beta-2-agonist +/− ICS), and 40% had received pulmonary rehabilitation within the past 2 years. The severity of breathlessness increased with increasing airflow limitation (r = 0.49, *p* < 0.0001) whereas other clinical characteristics (age, gender, BMI, smoking status) were not associated with breathlessness severity.

**Table 1:** Patients’ characteristics according to their breathlessness status

Patients’ characteristics	Whole cohort (*N* = 120)	Patients with severe breathlessness (mMRC ≥3) (*N* = 64)	Patients with Chronic breathlessness (*N* = 41)
Age (years) mean +/−SD	66 +/− 6	64 +/−4	64 +/− 4
Sex			
Male N (%)	69 (57%)	40 (62%)	26 (63%)
BMI (kg.m^2^) mean +/− SD	29 +/− 4	24 +/−2	25 +/−2
BMI > 30 kg.m^2^ N (%)	52 (43%)	27 (42%)	14 (34%)
Smoking status			
Non smoker N (%)	2(2%)	2 (3%)	2 (5%)
Former smoker N (%)	95 (79%)	47 (73%)	30 (73%)
Current smoker N (%)	23 (19%)	15 (24%)	9 (22%)
Smoking (Pack/year) mean +/−SD	40 +/− 12	38+/−8	40+/− 11
FEV1 (% pred) mean +/− SD	47 +/− 13	29 +/− 15*	26 +/− 9*
GOLD spirometric grade			
1 N (%)	3 (3%)	1 (1%)	1(2%)
2 N (%)	47 (39%)	16 (25%)*	5(12%)*
3 N (%)	18 (15%)	5 (8%)	2 (5%)
4 N (%)	52 (43%)	42 (66%)*	33(81%)*
Breathlessness: mMRC scale			
0 N (%)	3 (2%)	0	0
1 N (%)	16 (13%)	0	0
2 N (%)	37 (31%)	0	0
3 N (%)	39 (32%)	39 (61%)*	18 (44%)*
4 N (%)	25 (21%)	25 (39%)*	23 (56%)*
Frequent exacerbations (≥2/year) N (%)	49 (41%)	30 (47%)	20 (49%)

*BMI* Body mass index, *COPD* Chronic obstructive pulmonary disease, *FEV1* Forced expiratory volume in 1 s, *GOLD* Global initiative for obstructive lung disease, *mMRC* Modified Medical Research Council, data were expressed as mean +/− SD, * *p* ≤ 0.05, in comparison with the whole cohort

Patients with severe breathlessness (*n* = 64) exhibited more suspected anxiety and depression symptoms than those with non-severe breathlessness (respectively *p* = 0.003 and *p* = 0.001). HAD scale found quiet high proportions of patients with suspected anxiety (34%) or depression (30%) in patients with severe breathlessness. In terms of treatments targeting these symptoms, 27% of patients with positive HAD score received an anxiolytic and 16% an antidepressant. Patients with HAD-A score ≤ 10 or HAD-D score ≤ 10 didn’t have prescription of, respectively, anxiolytics or antidepressant.

Among the 64 patients with severe breathlessness (mMRC ≥3), 41 patients (64%) had chronic breathlessness. Of these, 17 patients still experienced breathlessness despite pulmonary rehabilitation. Chronic breathlessness moderately correlated with anxiety (r = 0.27, *p* = 0.023), and depression (r = 0.5, *p* < 0.0001) and inversely correlated with quality of life, measured by different scales (total SGRQ score = 73+/− 20, r = − 0.69, p < 0.0001, ADL score = 22+/− 10, r = 0.64, p < 0.0001). There was no difference in comorbidities except for anxiety, depression and osteoporosis between patients with chronic breathlessness or not (Additional file 1: Table S1). There was no link between chronic breathlessness and obesity or cardiovascular disease.

Among patients suffering from chronic breathlessness with suspected anxiety (HAD-A score > 10), only 20% had anxiolytics. Among those with suspected depression (HAD-D score > 10), only 13% received antidepressants.

Finally, among patients with chronic breathlessness, none had systemic opioids prescribed to treat breathlessness.

### Pulmonologists

In parallel, pulmonologists of the same geographical region were asked to respond to an on-line questionnaire on breathlessness management. Forty six pulmonologists (52%) among 88 working in the Lorraine region answered to the questionnaire. Among responders, 63% were male, with a mean age of 45 years; 35% worked in university hospital, 30% in general hospital, 24% had private practice, and 11% worked in general hospital and had private practice. Sixty three per cent were experienced pulmonologists; i.e. with over 10 years of practice. The physicians recommended mostly (for 94% of them) pulmonary rehabilitation to treat breathlessness in COPD and half of them believed that opioids have a role in treating dyspnea in stable COPD patients especially for hospitalized patients (48%) compared to out-patients (24%). Concerning medications (antidepressant and anxiolytics), 46% of the doctors declared to prescribe benzodiazepines against breathlessness for hospitalized patients and 13% for out-patients (Table 2).

**Table 2:** Pulmonologists’ willingness to prescribe specific treatments for breathlessness in COPD patients

	Outpatient N (%)	Hospitalized patients N (%)	Palliative care N (%)
Medications			
Systemic morphine	11 (24%)	22 (48%)	42 (91%)
Nebulised morphine	-	2 (4%)	9 (20%)
Benzodiazepines	6 (13%)	21 (46%)	32 (70%)
Oxygen	8 (17%)	14 (30%)	24 (52%)
Nebulised furosemide	-	2 (4%)	-
None	27 (59%)	-	-
Non-pharmacologic interventions			
Pulmonary rehabilitation	44 (96%)		
Sport	27 (59%)		
Relaxation	10 (22%)		

## Discussion

The goals of this study were to determine the clinical characteristics of COPD patients with severe and/or chronic breathlessness and their treatments used in a real-life cohort. Since this cohort was performed in a tertiary care hospital and to have a wider view, we also investigated the knowledge and beliefs of pulmonologists, working in the same region, regarding the management of severe breathlessness with a special interest in the use of opioids and pulmonary rehabilitation for COPD patients. Indeed chronic breathlessness shares common characteristics with chronic pain but is less likely to be recognised by physicians [18].

In our study the prevalence of severe breathlessness (mMRC≥3) and chronic breathlessness severe breathlessness that persists despite optimal treatment of the underlying pathology) was comparable to previous data [1]. Sundh et al. defined maximal treatment as triple inhaled therapy and physiotherapy [19]. However ICS have no or marginal effects on breathlessness. Therefore we considered that an optimal pharmacological treatment for breathlessness consisted in LABA and LAMA [2, 17]. Besides, we separated chronic breathlessness despite pulmonary rehabilitation from chronic breathlessness without pulmonary rehabilitation. Of note, in the study by Sundh et al., disabling breathlessness was defined as a mMRC score ≥ 2 [19], whereas in our work severe breathlessness is defined by a mMRC score ≥ 3.

The severity of breathlessness was associated with lung function impairment but there was no specific clinical characteristic associated with chronic breathlessness in terms of other symptoms, exacerbation rate, and comorbidities, except for increased symptoms of anxiety and/or depression as already described [20] and osteoporosis. The link between the severity of breathlessness and osteoporosis may be the consequence of a decreased physical activity in these patients [21].

Cardiovascular comorbidities were defined by declarative way, and/or if the patient underwent a cardiologic follow-up, and/or if he received specific cardiovascular medications (at least beta-blockers or angiotensin-converting enzyme inhibitors). However, systematic cardiovascular exploration was not mandatory for each patient, which could explain the absence of link between breathlessness and cardiovascular diseases, which are also risk factors for this symptom.

Although pulmonary rehabilitation has been shown to improve breathlessness and quality of life [22], this treatment was underutilised in this COPD cohort, as previously reported [6, 7, 9]. As GOLD 2018 update described, the main problem of pulmonary rehabilitation is limited “access”. However, the proportion of patients receiving pulmonary rehabilitation is well beyond what is usually described [23], which may be due to the setting of the study (tertiary care centre). Besides, in France, pulmonary rehabilitation is covered by insurance companies and in this specific region, the health network around pulmonary rehabilitation is well organized (150 physiotherapists and rehabilitation organized in 7 general hospitals and 2 private hospitals, added to 4 territorial health networks).Further, our study clearly shows that some patients continue to experience chronic breathlessness despite pulmonary rehabilitation. This must be in part due to the fact that the effect is not persistent after 2 years.

A striking result of our study is the complete lack of use of opioids despite international recommendations [17] and previous studies showing that opioids reduce severe dyspnea in advanced COPD with stable clinic condition. By contrast, the results of the questionnaire survey demonstrate a high willingness to prescribe opioids for the off-licence indication of chronic breathlessness in COPD, as in other study [24, 25]. In our study, 30% of the 46 responding physicians have expressed a reluctance to prescribe opioids in the context of COPD. Sixty-six percent of them justify this reluctance because they are afraid of respiratory side effects such as respiratory depression. In the literature, reluctance to prescribe opioids ranges from 20 to 55% [25–27]. Another study performed in practices of young Australian pulmonologists also found that less than 25% of them have already prescribed opiods [24]. Indeed, in our study, young doctors seem more reluctant than their elders to prescribe opioids in this context.

Regarding suspected anxiety and/or depression (HAD score > 10), our results clearly show an underuse of specific medications including anxiolytics and antidepressants, especially in patients with chronic breathlessness. GOLD committee recommends that, in patients with COPD, both disorders should be treated according to usual guidelines, as there is no evidence that anxiety/depression should be treated differently in the presence of COPD [17]. However, we used the HAD scale to screen for anxiety or depression but there was no intervention of a psychiatrist to confirm these disorders.

In this prospective cohort, we examined well characterized patients using tools validated in COPD. This is the only study describing patients’ characteristics and pulmonologists’ attitudes simultaneously. However, this study has some limitations. It uses a cross-sectional monocentric design and the questionnaire results are, by definition, declarative. Another limitation is the low number of patients with breathlessness and particularly with severe breathlessness.

Finally, although opioids seem to be an interesting tool for breathlessness relief in COPD patients, randomized controlled trials are needed to determine the real clinical benefit and the most suitable target population [28]. Long-term opioid use is cautiously recommended as tight surveillance is needed and their respiratory side effects can be crippling in chronic respiratory disease, particularly in older patients [10, 11] and in patients with chronic respiratory insufficiency [28]. That could explain why many physicians remain reluctant to prescribe opioids especially due to fear of respiratory adverse effects [28] and the off-licence prescription of these medications.

## Conclusion

Management of breathlessness and frequent comorbidities, such as symptoms of anxiety and/or depression in COPD is insufficient despite pulmonologist’s knowledge and willingness. Promoting pulmonary rehabilitation and symptomatic pharmacological treatments might be a key element in the overall management of COPD with a role for opioids use with a tight surveillance.

## Additional file

Additional file 1: **Table S1.** Comorbidities associated with non chronic breathlessness versus chronic breathlessness despite pulmonary rehabilitation. Univariate analysis. (DOCX 15 kb)

## Abbreviations

BMI: Body Mass Index
COPD: Chronic Obstructive Pulmonary Disease
FEV1: Forced Expiratory Volume in 1 s
GOLD: Global initiative for Obstructive Lung Disease
HAD: Hospital Anxiety and Depression scale
mMRC: Modified Medical Research Council
OSAS: Obstructive Sleep Apnea Syndrome.
