# Supplementary material for: Prevalence and management of chronic breathlessness in COPD in a tertiary care center
Source: BMC Pulm Med. 2019 May 16;19:95. doi: 10.1186/s12890-019-0851-5 (PMC6524222; doi:10.1186/s12890-019-0851-5)
Supplement: Supplementary file 1 — Table S1. Comorbidities associated with non chronic breathlessness versus chronic breathlessness despite pulmonary rehabilitation. Univariate analysis. (DOCX 15 kb) [file 12890_2019_851_MOESM1_ESM.docx]

Additional file 1: **Table S1** Comorbidities associated with non chronic breathlessness versus chronic breathlessness despite pulmonary rehabilitation. Univariate analysis.

| **Comorbidities** | **No chronic breathlessness**  **N=79** | **All chronic breathlessness**  **N=41** | **Chronic breathlessness despite pulmonary rehabilitation**  **N=17** |
| --- | --- | --- | --- |
|  | N (%) | N (%) | N (%) |
| Arterial hypertension  Coronary artery disease  Arrhythmia  Diabetes  Left heart failure | 40 (51%)  14 (18%)  19 (24%)  14 (18%)  12 (15%) | 20 (49%)  3 (7%)  6 (15%)  7 (17%)  7 (17%) | 6 (35%)  1 (6%)  3 (18%)  1 (6%)  6 (35%) |
| Obesity  Pulmonary hypertension  OSAS  Pulmonary embolism  Lung cancer  Osteoporosis  Chronic pain | 36 (46%)  5 (6%)  22 (28%)  3 (4%)  2 (3%)  5 (6%)  15 (19%) | 16 (39%)  7 (17%)  12 (29%)  1 (2%)  2 (5%)  **10 (24%) ***  3 (7%) | 4 (24%)  2 (12%)  4 (24%)  0 (0%)  1 (6%)  **6 (35%) ***  1 (6%) |
| HAD anxiety > 10  HAD depression > 10 | 13 (17%)  8 (11%) | 15 (37%)  15 (39%) | **8 (47%) ***  **8 (47%) *** |

HAD = hospital anxiety and depression scale, OSAS = obstructive sleep apnea syndrome. Data were expressed as mean +/- SD, * p≤0.05, comparison with no chronic breathlessness
